# An Experiment and Simulation Study on the Tensile Behavior of Cotton Ring-Spun Yarn with Twisted Staple Fibers

**DOI:** 10.3390/ma19030560

**Published:** 2026-01-30

**Authors:** Xiaoshuang Xiong, Shuyang Wu, Lingyao Zeng, Jiacheng Zhou, Zhaochong Hou, Xiang Li, Mingzhang Chen, Chen Shen, Fei Fan

**Affiliations:** 1 Hubei Key Laboratory of Digital Textile Equipment, Wuhan Textile University, Wuhan 430200, China; xsxiong@wtu.edu.cn (X.X.); cshen@wtu.edu.cn (C.S.); ffan@wtu.edu.cn (F.F.); 2 Three-Dimensional Textile Hubei Engineering Research Center, Wuhan Textile University, Wuhan 430200, China; 3 School of Mechanical Engineering & Automation, Wuhan Textile University, Wuhan 430200, China; 2315373050@wtu.edu.cn (S.W.); zly2001zly@gmail.com (L.Z.); 4 Shandong Rifa Textile Machinery Co., Ltd., Liaocheng 252001, China; hzcxtt@163.com; 5 Key Laboratory of Safety of Hydrogen Energy Storage and Transportation Equipment, State Administration for Market Regulation, Beijing 100029, China; 6 Hubei Key Laboratory of Advanced Technology for Automotive Components, Wuhan University of Technology, Wuhan 430070, China; chenmingzhang@whut.edu.cn

**Keywords:** cotton ring-spun yarn, tensile properties, twist angle, friction coefficient, numerical simulation

## Abstract

This paper investigates the tensile behavior of cotton ring-spun yarn through experimental testing, numerical simulation, and theoretical calculation. Firstly, scanning electron microscope testing of the microscopic geometric morphologies of yarns was performed for the development of basic finite element (FE) models. Then, the influences of tensile speed and yarn length on the tensile properties of yarn were studied using tensile experiments. Numerical simulations were further performed to investigate the effects of yarn diameter, twist angle, and friction between fibers on the tensile modulus of yarn. Finally, a modified ‘rule-of-mixtures’ equation was proposed to effectively calculate the tensile modulus of yarn through incorporating the friction correction factor. The experimental results show that the tensile modulus and strength of tested yarn are significantly affected by the yarn structure and are not sensitive to the yarn length and tensile speed. Furthermore, the tensile moduli of yarns obtained from the numerical simulations show a good fitting accuracy with those obtained from experimental tests when the friction coefficient is set to 0.5 in the FE models. The simulation results show that the twist angle and friction coefficient are two key factors affecting the tensile modulus of yarn. The modified ‘rule-of-mixtures’ equation presents better accuracy for the calculation of the tensile modulus of yarn compared with the traditional ‘rule-of-mixtures’ equation, which can be used to replace the FE modeling and simulation and reduce the computational cost. This work will provide a deeper understanding of the mechanical properties of cotton ring-spun yarns and enhance their application in the textile industry.

## 1. Introduction

The cotton textile industry is a vital pillar and livelihood industry in many countries around the world, especially in developing countries such as China, India, Pakistan and Bangladesh [1,2,3,4]. Cotton ring-spun yarn made from twisted staple fibers is widely used in the fields of clothing, home textiles, and industrial fabrics [5,6,7,8]. The macroscopic structural morphology, such as the twisting angle, diameter, etc., and the microscopic fiber arrangement have a great effect on the mechanical properties of cotton ring-spun yarn and further affect the performance of the weaving process [9,10]. In order to better guide the technological process of spinning and weaving and obtain high-performance textile fabrics, it is of great value to investigate the relationship between the microscopic and macroscopic structure and mechanical properties of cotton yarn [11,12].

Conventional cotton yarn is a conventional twisted yarn in which the natural cotton fibers are of a short length, known as staple fibers in the textile industry. Moreover, the cotton fibers in the twisted yarn are held together by the fiber-to-fiber friction derived from the helical fiber path (i.e., twist) during the ring-spinning process [13]. In order to study the mechanical properties of conventional twisted yarn, experimental testing, numerical simulation, and theoretical analysis are used to consider the characteristics of the microscopic and macroscopic structure of yarn. The spinning method determines the macroscopic structure of the yarn, and the microscopic yarn structure is determined by the properties of the raw materials and the twisting method [14,15,16]. Cui et al. conducted experimental tests to investigate the number of self-twists in different yarns and found that the value of the twist angle was proportional to the ratio of fiber torsional stiffness to bending stiffness [17]. They concluded that the wool and acrylic fibers were more suitable for use with the self-twist spinning method because of the greater self-twist value. Ma et al. studied the mechanical properties and morphological changes in Xinjiang long-staple cotton fibers during the tensile process and investigated the influence of fiber diameter, fiber length, and tensile speed on the mechanical properties and the fracture mechanism of cotton fibers [18]. Yang et al. further found that the separation of fiber fibrils and the fracturing of individual fibrils or fiber bundles were two main mechanisms for cotton-fiber tensile breakage [19]. It can be concluded that the tensile strength and stiffness of cotton fiber as the raw material of cotton yarn are influenced by the microscopic multi-walled structure, the fiber diameter, the fiber length, and the tensile test conditions, which then affect the final mechanical and fabrication properties of cotton yarn. However, many experimental factors, such as the laboratory temperature and humidity, the tester’s operation skills, the test instruments, and the setting parameters, have an effect on the test results of the mechanical properties of cotton fibers and yarns. Therefore, it is of great importance to develop a simulation model to investigate the mechanical properties of cotton yarn for reducing experimental errors and costs [20,21,22].

Ge’gauff firstly proposed a geometrical model of twisted yarns with a coaxial helix structure, which had been widely adopted as a basic model in modern yarn structural mechanics analysis [23]. Gu and Miao investigated the influence of twist on the tensile properties of short-fiber yarns and found that the tensile strength was increased first with the increase in twisting angle and then decreased due to the balance between the fiber obliquity in relation to the yarn axis and the fiber-to-fiber friction [24]. Miao et al. further proposed a theoretical calculation method to analyze the effect of twist angle of fibers on the yarn surface on the elastic modulus of yarn [25]. The above work of Miao et al. on the investigation of the tensile properties of yarn are based on the assumption that the mechanical properties of fibers in the yarn were the linear elasticity. Xiong et al. further proposed a theoretical calculation approach by introducing the transversely isotropic material property of fiber to study the effect of surface twist angle of yarn on the effective elastic constants of twisted yarn [26]. They found that the longitudinal modulus was dramatically decreased with the increase in twist angle, while all other elastic constants were increased slowly with the increase in twist angle. All the above theoretical analysis models are based on the classical coaxial helix geometrical model of twisted yarn and only take into account the mechanical properties of fibers and twist angle, while the friction between fibers is neglected. However, the friction between fibers is an important factor affecting the tensile behavior of cotton yarn, in which the cotton fibers are closely twisted together by the fiber-to-fiber friction.

In order to further investigate the interaction of fibers in the twisted yarn during different load conditions, finite element (FE) modeling has been widely adopted [27,28,29,30,31]. Djaja et al. firstly developed an FE model of yarn with a concentric cylindrical geometry to predict the mechanical constants through precisely defining the stiffness matrix of each element by considering the oriented helical distribution of fibers [32]. An FE model considering the geometry structure and the non-linear filament properties was proposed by Sriprateep et al. to simulate the tensile behavior of multi-filament twisted yarns with the same numbers of layers and different twist angles, in which the friction between filaments was assumed to be high, and the interface between the filaments was set as bonded [33]. Therefore, the relative sliding of filaments during the tension deformation was neglected in their simulation. Based on the proposed FE simulation method, Sriprateep further investigated the stress–strain curves of a wide range of man-made filament yarns from nine material types and compared the prediction results with the stress–strain curves of experimental results [34]. Considering the actual microstructure of polyester staple spun yarn, Zhang et al. proposed an innovative FE modeling approach for analyzing the dynamic behavior of fibers in the staple spun yarn based on the geometrical model derived from X-ray micrographs. Based on the proposed FE model, they conducted an in-depth study on the influence of friction coefficient on the tensile properties of yarns and concluded that a low friction coefficient of 0.1 caused a significant reduction in fiber-to-fiber friction, while the fiber-to-fiber friction was not affected when the friction coefficient increased from 0.3 to 0.5 [35]. Although the above FE simulations have widely investigated the single effect of twist angle and friction coefficient between fibers on the mechanical properties of yarn, the coupling effects of twist angle and friction coefficient are rarely studied, which are the key characteristics of cotton ring-spun yarn.

In this paper, finite element models of cotton ring-spun yarns were developed based on the microscopic structural characteristics of yarns. Tensile tests for yarns were conducted to verify the accuracy of the FE simulations. Based on the FE models, the effects of twist angle, yarn diameter and friction coefficient on the tensile properties of yarn were investigated. Meanwhile, a modified model based on the ‘rules of mixtures’ for predicting the tensile modulus of twisted single yarns was proposed to replace the FE modeling and simulation and reduce the computational cost, considering the coupling effects of twist angle and friction coefficient between fibers. This work provides a theoretical guidance for the performance analysis and industrial application of cotton ring-spun yarn.

## 2. Geometric Model Construction

### 2.1. Characterization of Yarn Geometric Morphology

**Figure 1:**
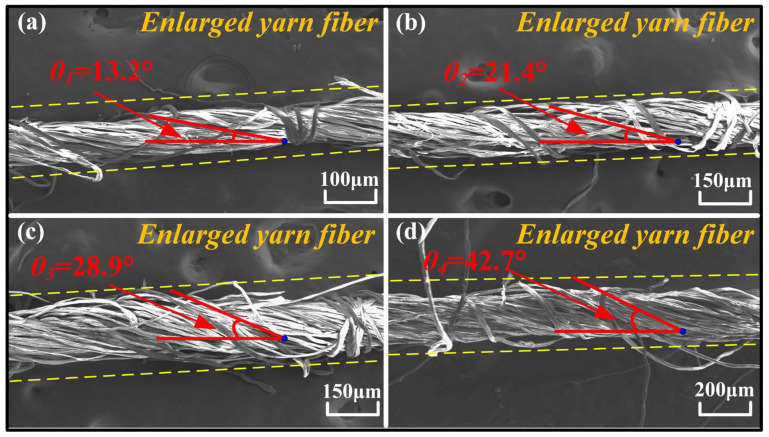
SEM images of 4 types of yarns: (**a**) Y1, (**b**) Y2, (**c**) Y3, and (**d**) Y4.

Four types of cotton staple ring-spun yarns provided by Shandong Rifa Textile Machinery Co., Ltd. (Liaocheng, China) were adopted in the experimental test and finite element simulation, of which the basic parameters are presented in Table 1. In order to build the precise geometric model of the yarns, a scanning electron microscope (Hitachi SU5000, Hitachi, Ltd., Tokyo, Japan, 10 kV) was used to characterize the geometric morphology of four types of yarns, and all yarn samples underwent gold-spraying treatment to enhance their conductivity before the scanning electron microscope (SEM) testing. The microscope images of four types of cotton staple ring-spun yarns are presented in Figure 1. As shown in Figure 1, the fibers making up the four types of yarns predominantly follow an ideal spiral trajectory and lead to the tight structures of the yarns, which are the typical structure characteristics of yarns manufactured by the ring-spinning method. Through the statistical analysis, the average diameter of single cotton fibers in all four yarns is about 15 μm. The average twist angles of the four yarns are 13.2°, 21.4°, 28.9°, and 42.7°, respectively, while the average diameters of the four yarns are 0.112 mm, 0.146 mm, 0.169 mm and 0.208 mm, respectively. Furthermore, the four types of yarns are defined as Y1, Y2, Y3 and Y4 according to the rank of their average diameter.

### 2.2. Construction of Yarn Geometric Model

**Figure 2:**
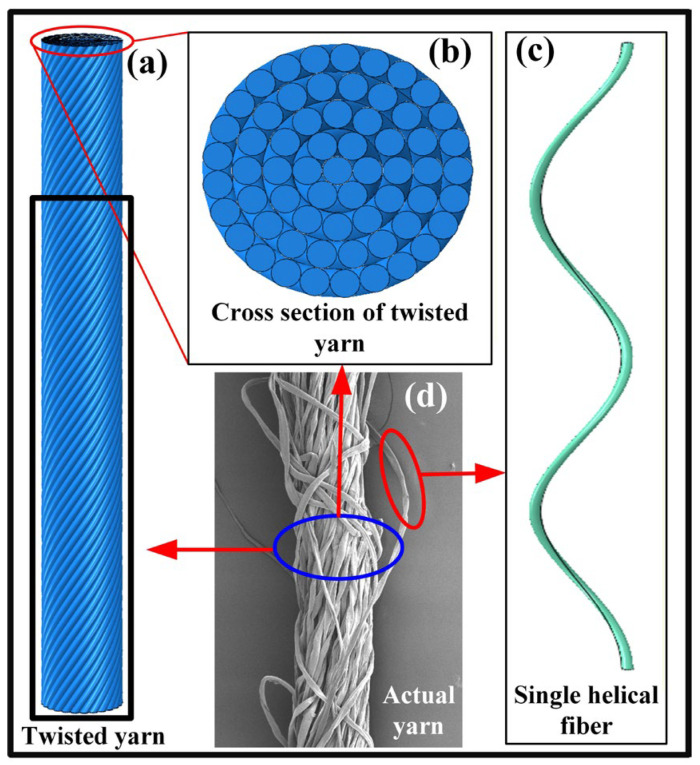
Schematic diagram of yarn geometric model: (**a**) twisted yarn, (**b**) cross-section of twisted yarn, (**c**) single helical fiber, and (**d**) actual yarn.

**Figure 3:**
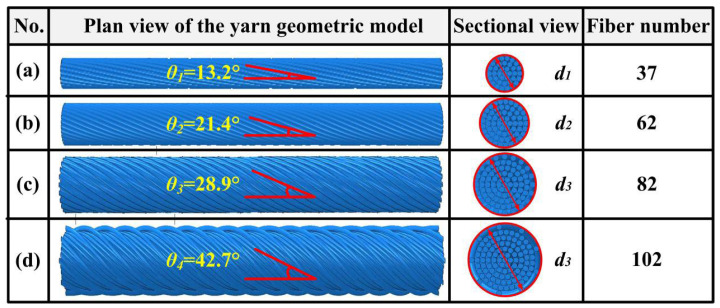
Four basic types of yarn geometric models: (**a**) S1, (**b**) S2, (**c**) S3, and (**d**) S4.

Based on the microstructure analysis of cotton ring-spun yarn presented in Section 2.1, an idealized geometry of yarn consisting of helical fibers (Figure 2a) was developed to represent the actual yarn (Figure 2d) [30,33]. In the idealized geometric model, the fiber at the center follows the straight line of the yarn axis, while the helix angles of fibers out from the center gradually increase to keep a constant number of twist turns in all the layers [33]. That is to say, all fibers being concentric have the same pitch. As shown in Figure 2b,c, the cross-sectional shape of fibers is chosen to be approximately circular, and the number of fibers increases proportionally from the center to the outer layer, ensuring the uniform density of the packing of fibers in the yarn. Furthermore, the fibers with a diameter of 15 μm in the yarn are arranged closely, but without the interference or penetration. Through changing the number and pitch of layers of the yarn, the yarn model with different diameters and twist angles can be obtained. In order to verify the accuracy of the next numerical simulations, four basic types of yarn geometric models with different twist angles, yarn diameters, and yarn twist turns are developed as listed in Table 2, of which the geometric structures correspond to the geometric morphologies of cotton ring-spun yarns shown in Figure 1. Furthermore, the four types of yarn geometric models are defined as S1, S2, S3 and S4 with a similar simulation length according to the rank of their average diameters, which are presented in Figure 3. Fiber migrations, variable twist angles, variations in the packing density across the cross-section, and discontinuous fiber lengths are ignored in the proposed yarn geometric models, which may cause an increase in predicted values of the tensile moduli of yarns.

## 3. Development of FE Models and Experimental Apparatus

### 3.1. Experimental Tests

**Figure 4:**
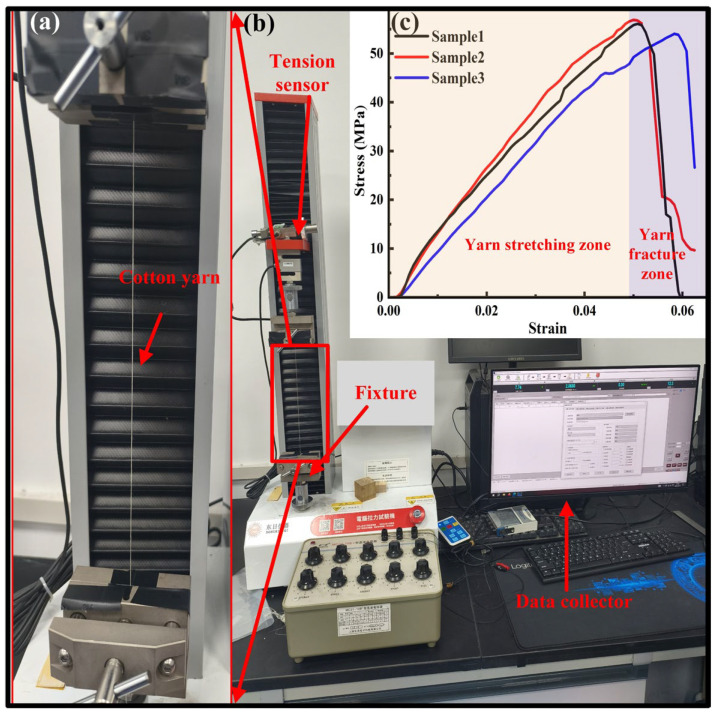
Tensile testing: (**a**) tested yarn, (**b**) tensile testing machine, and (**c**) tensile stress–strain of yarn.

A universal tensile testing system (DR-507ASQ, Dongri Instrument Co., Ltd., Dongguan, China) as shown in Figure 4 was used to investigate the tensile behaviors of four types of cotton ring-spun yarns (Y1, Y2, Y3 and Y4) listed in Table 1, according to the ASTM D2256 standard test method [36]. In order to investigate the effects of yarn length and tensile speed on the tensile strength and modulus of yarn, five gauge lengths (50 mm, 100 mm, 150 mm, 200 mm, and 250 mm) and three tensile speeds (1 mm/s, 2 mm/s, and 3 mm/s) for yarns (Y1, Y2, Y3 and Y4) were chosen in the experimental testing [18]. As presented in Figure 4a, the yarn must be adjusted to a slightly tensioned state before the tensile testing. Furthermore, each testing type of gauge length and tensile speed was repeated 20 times to obtain the statistical tensile curves because of the inconsistent performance of cotton yarn as a natural material, as shown in Figure 4c. Furthermore, the room temperature was 25 °C and the relative humidity was 65% during the tensile testing.

### 3.2. Establishment of Simulation Models

**Figure 5:**
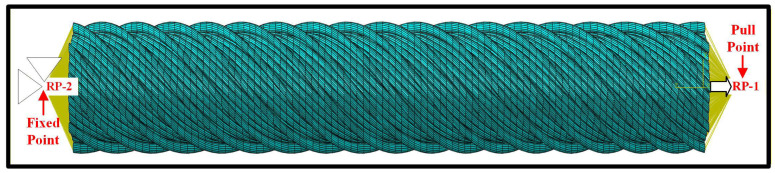
Boundary conditions for the finite element model of yarn.

In order to predict the effects of the twist angle and diameter of yarn and the frictional contact between the fibers on the tensile properties of cotton ring-spun yarns, the finite element (FE) models of yarns were developed using the software Abaqus 2024/Explicit solver, according to the ASTM D2256 standard test method [36]. The mechanical properties of the cotton fibers used in the simulation were defined as anisotropic materials as listed in Table 3, which were implemented in the FE models using the discrete definition method. Furthermore, the density of the cotton fiber was set to 1.5 × 10^3^ kg/m^3^. Subsequently, the cotton fiber was meshed with 8-node solid elements (C3D8R), and each fiber consisted of 4800 elements. The setting of boundary conditions for the tensile simulation is presented in Figure 5. As shown in Figure 5, the upper and lower cross-sections of fibers were coupled with the center points of the top and bottom sides of yarn, respectively. All degrees of freedom of the center point (RP-2) on the bottom side were fixed, while the degrees of freedom of the center point (RP-1) on the top side were fixed except the translational freedom in the tensile direction. The displacement was applied to the RP-1 along the axial direction of yarn to simulate the tensile process and the uniform tensile speed was set to 1 mm/s. In the experiment and simulation, the effective stress of tensile load is calculated by dividing the tensile load by the cross-sectional area of the yarn. Finally, the effects of yarn twist angle, yarn diameter and friction coefficient between the contacting surfaces of fibers on the tensile properties of yarn were investigated based on the FE models.

## 4. Results and Discussion

### 4.1. Analysis of Experimental Results

**Figure 6:**
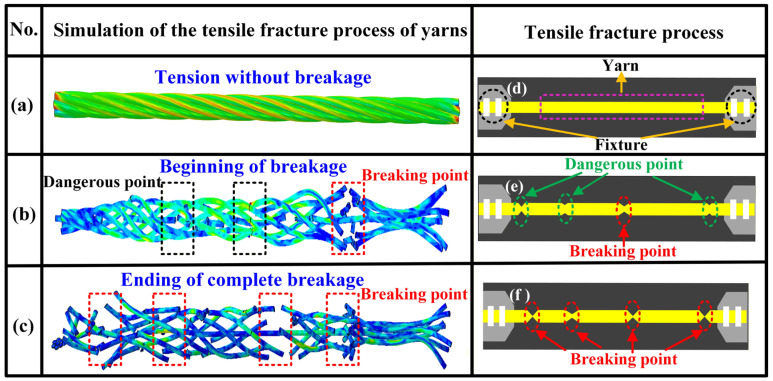
Illustration of tensile fracture process of the yarn: (**a**) stress distribution of yarn in the state of tension without breakage, (**b**) stress distribution of yarn in the state of beginning of breakage, (**c**) stress distribution of yarn in the state of ending of complete breakage, (**d**) tested yarn in the state of tension without breakage, (**e**) tested yarn in the state of beginning of breakage, and (**f**) tested yarn in the state of ending of complete breakage.

Figure 6 presents the tensile fracture process of the yarn. Figure 6a–c show the stress distribution map of the yarn at different stages of the tensile breakage process, including: the tension without breakage, the beginning of breakage, and the ending of complete fracture. During the tensile process, the breakage initially occurs in the area with the highest stress and the weakest structure and then appears simultaneously in many other areas. Figure 6d–f briefly illustrate the failure mechanism of the tested yarn. The two ending sides of yarn are clamped by the fixtures shown in Figure 6d, and the midpoint of yarn frequently serves as the primary failure location while the secondary failure points remain distributed along the ends shown in Figure 6e. When all secondary failure points are stretched and broken, the yarn completely fails, as presented in Figure 6f.

**Figure 7:**
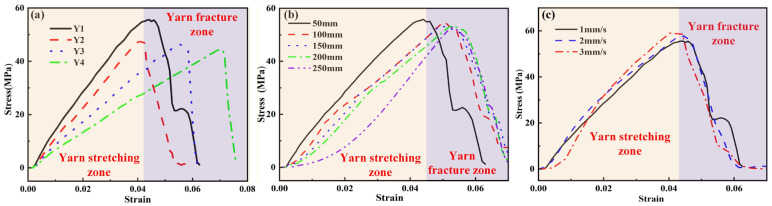
Stress–strain curves for four types of yarn samples (Y1, Y2, Y3, and Y4) under different testing conditions: (**a**) tensile condition: a yarn length of 50 mm and tensile speed of 1 mm/s, (**b**) yarn (Y1) at a tensile speed of 1 mm/s for five different yarn lengths, and (**c**) yarn (Y1) at a length of 50 mm for three different tensile speeds.

Figure 7 displays the stress–strain curves for four types of yarn samples (Y1, Y2, Y3, and Y4) under different testing conditions. It can be obviously observed that all stress–strain curves consist of a yarn stretching zone and yarn fracture zone. The stress of all curves is not increased and about zero with a strain of about 0.005, because the yarns are stretched from their natural state to the tensile state. Furthermore, the stress slightly increases to the maximum value in the yarn stretching zone and then declines quickly in the yarn fracture zone, indicating the rapid failure of yarn. As shown in Figure 7a, the yarn with a smaller diameter and lower twist angle exhibits a higher tensile modulus and strength. Similar results have been published in the work of Sriprateep et al. [33,34]. This occurs because the smaller diameter and lower twist angle result in a denser internal structure and enhance the structural stability of yarn. It can be found from Figure 7b that the yarn length has a slight influence on the yarn strength, and all samples are fractured at a similar tensile strength of about 50 MPa. When the yarn length increases from 50 mm to 250 mm, the slope of the stress–strain relationship slightly decreases, indicating a mild decrease in tensile modulus. For the tested yarn with a longer length, the probability of inherent defects such as impurities, weak points, and irregularities increases and the cross-linking between the fibers increases, which synthetically causes a slight decrease in tensile strength and modulus. The influence of different tensile speeds on the tensile stress–strain curve of yarn is presented in Figure 7c. When the tensile speed increases from 1 mm/s to 3 mm/s, both the tensile modulus and strength mildly increase within 5%. It is obvious that the tensile speeds of 1 mm/s to 3 mm/s in the experimental tests are low tensile speed conditions and the cotton yarn exhibits a linear elastic behavior. Therefore, the elastic modulus of cotton fiber is only taken into account in the numerical model, which only simulates the tensile behaviors of yarn under the low tensile speed of 1 mm/s and focuses on the investigation of the effect of yarn diameter, twist angle, and friction coefficient.

**Figure 8:**
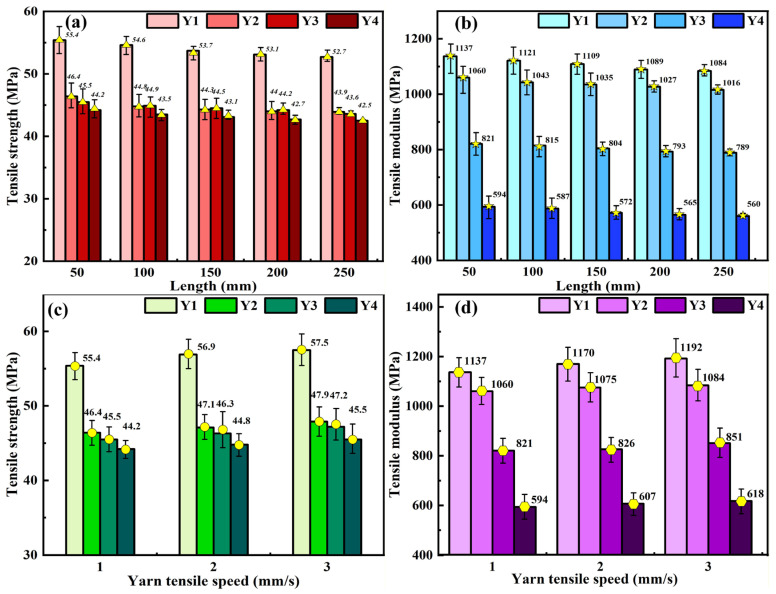
Statistics on tensile strength and tensile modulus for four yarn samples (Y1, Y2, Y3, and Y4) under different test conditions: (**a**) tensile strength at five different lengths with an identical tensile speed of 1 mm/s, (**b**) tensile modulus at five different lengths with an identical tensile speed of 1 mm/s, (**c**) tensile strength at three different tensile speeds with an identical yarn length of 50 mm, and (**d**) tensile modulus at three different tensile speeds with an identical yarn length of 50 mm.

Figure 8 further presents the statistical analysis of tensile strength and modulus of four types of yarn samples (Y1, Y2, Y3, and Y4) under different yarn lengths and tensile speeds. It can be concluded again that the yarn (Y1) with the lowest twist angle of 13.2° has the highest tensile strength and modulus under the same yarn length and tensile speed, while the yarn (Y4) with the largest twist angle of 42.7° has the lowest tensile strength and modulus. Meanwhile, it can be further found that the effects of yarn length and tensile speed on the tensile strength and modulus of yarn are unobvious. Therefore, we developed four types of yarn models with a similar yarn length of about 2 mm (Table 2 and Figure 3) and a tensile speed of 1 mm/s in the finite element simulations and focused on the investigation of the effects of yarn diameter, twist angle, and friction coefficient.

### 4.2. Verification for Numerical Simulation Model

**Figure 9:**
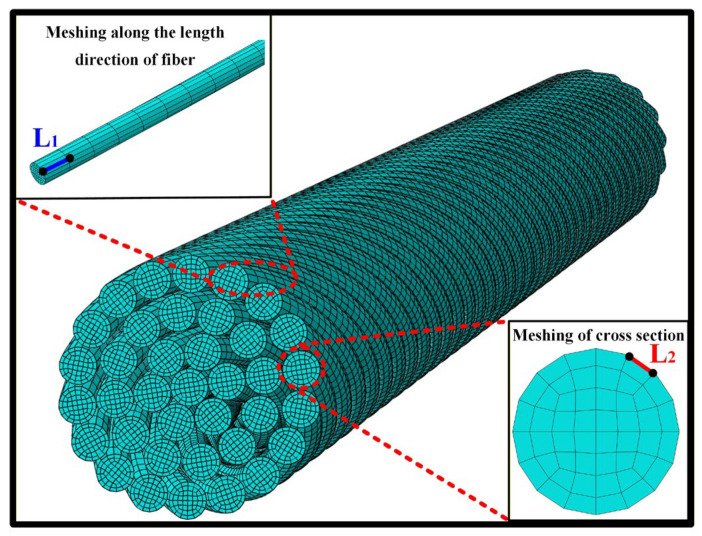
Schematic diagram of mesh density of yarn.

**Figure 10:**
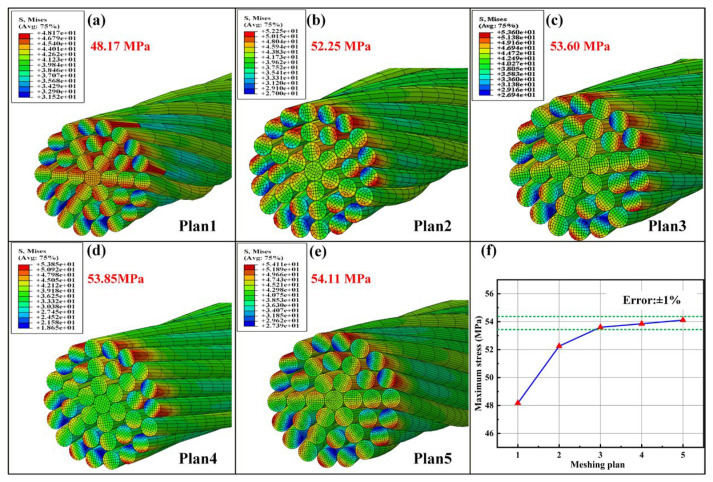
Verification for the mesh convergence: (**a**–**e**) are the maximum von Mises stress distributions of yarn with different element meshing sizes (simulation conditions: S1, a friction coefficient of 0.5, and tensile speed of 1 mm/s) and (**f**) mesh convergence.

In order to verify the influence of mesh size on the stress distribution of yarn, five element meshing plans of cotton fibers as listed in Table 4 are designed and adopted through changing the size of elements along the length direction of fiber and in the cross-section of fiber, as shown in Figure 9. Figure 10 presents the verification for the mesh convergence. It can be obviously observed that, when the size of elements along the length direction of fiber and in the cross-section of fiber respectively reach about 20 μm and 2.9 μm, the convergence of the maximum von Mises stress of fiber is obtained, and then meshing plan 3 is chosen for the mesh of yarn in the simulations. In the end, the cotton fiber of yarn was meshed with 8-node solid elements (C3D8R), and each fiber consisted of 4800 elements.

**Figure 11:**
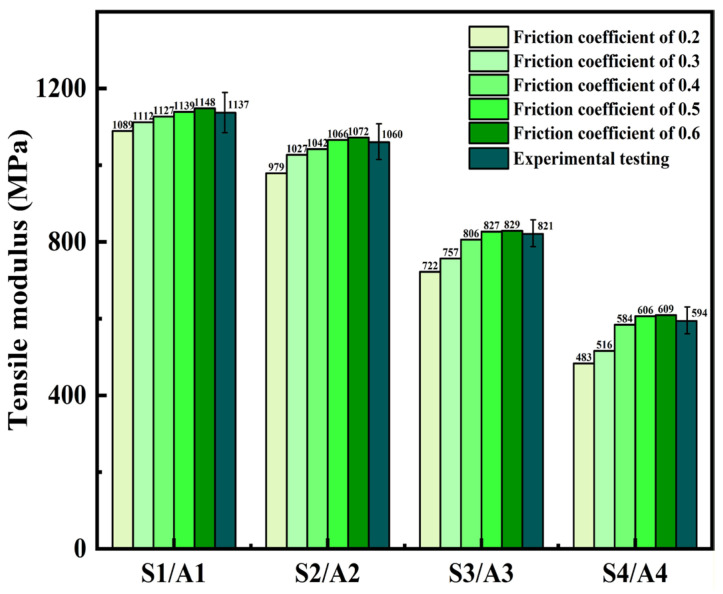
Comparison between simulation results and experiment results (tensile condition: a yarn length of 50 mm and tensile speed of 1 mm/s) of tensile modulus of four types of yarn.

To investigate the relationship between the tensile properties of cotton yarns and their twist angle, diameter, and friction coefficient, numerical simulation models of twisted yarns were developed. Four simulated yarn models (S1, S2, S3, and S4) with different friction coefficients of 0.2 to 0.6 according to the literature were developed based on the structural parameters of four types of experimental yarn (Y1, Y2, Y3, and Y4) [35,38]. The effects of friction coefficient on the tensile modulus of all types of yarn obtained from the numerical simulations are compared with those from the experimental tests in Figure 11. From Figure 11, the simulation results of tensile modulus of all types of yarn with a friction coefficient of 0.5 are most consistent with the experimental results, and the maximum fitting error of them is less than 2%. Therefore, the friction coefficient of 0.5 will be chosen in the further numerical simulations for the stress analysis of different yarns.

**Figure 12:**
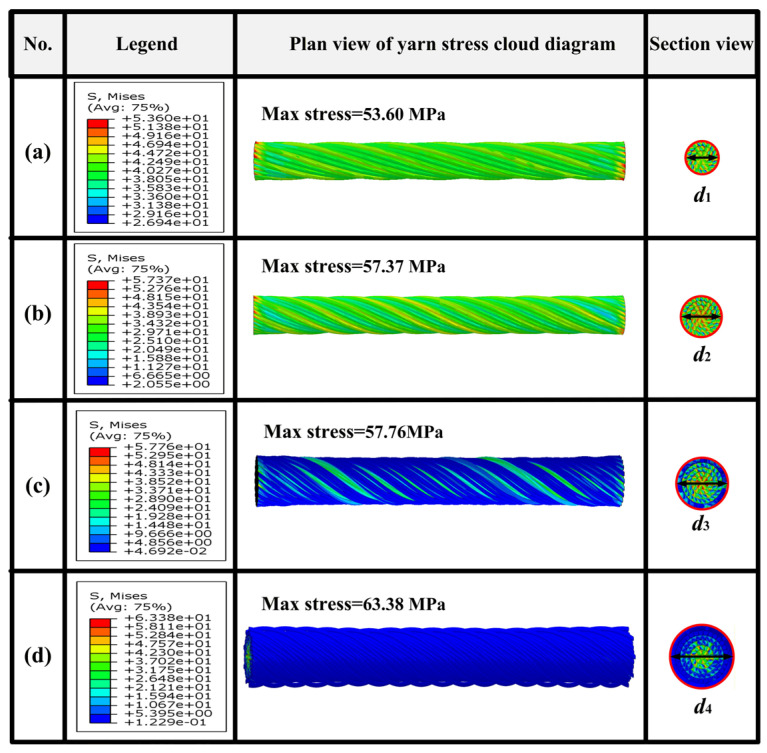
Stress characteristics of four types of simulated yarns: (**a**) S1, (**b**) S2, (**c**) S3, and (**d**) S4.

Figure 12 displays the stress distribution of the four simulated yarn models at a strain of 0.03. As seen in Figure 12, the stress is primarily concentrated in the central region of the cross-section of each type of yarn and gradually decreases to the outer layer. Furthermore, the maximum stress of fibers in each layer always occurs at the contact points between two fiber layers. Obviously, with the increase in yarn diameter, the stresses of fibers in the outer layer are significantly lower than those in the inner layer, while the stress distribution becomes more uneven.

**Figure 13:**
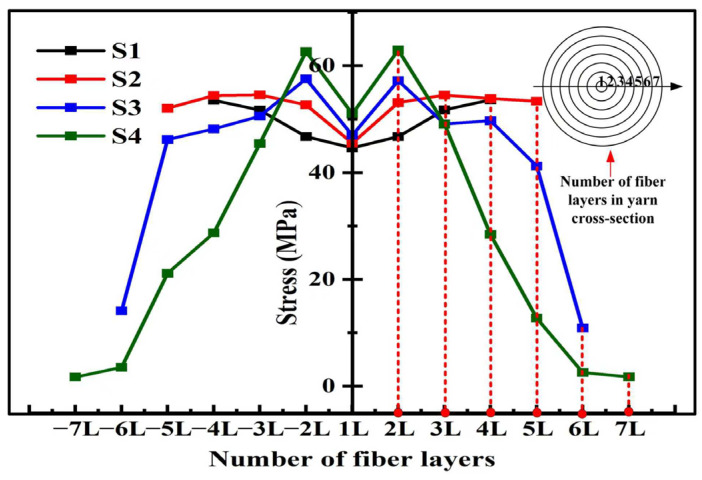
Maximum stress distribution from the inner layer to the outer layer in the cross-section of four types of simulated yarns (S1, S2, S3, and S4).

Figure 13 further displays the maximum stress distribution within each fiber layer of the four types of yarn. For yarns S1 and S2, the maximum stress occurs in the outer layer, whereas, for yarns S3 and S4, the maximum stress occurs in the second layer. The difference of maximum stress between different layers is not notable for yarns S1 and S2. The interactions between outer-layer fibers during stretching generate friction forces, causing the higher maximum stress of yarns S1 and S2 in the outer layers. The maximum stress for yarn S4 decreases sharply from the inner layer to the outer layer due to its larger twist angle and diameter, which cause the larger difference in strain between the inner and the outer layer under the tensile load. Therefore, it can be concluded that the more uniform stress distribution contributes to the higher tensile strength and modulus of yarns S1 and S2.

**Figure 14:**
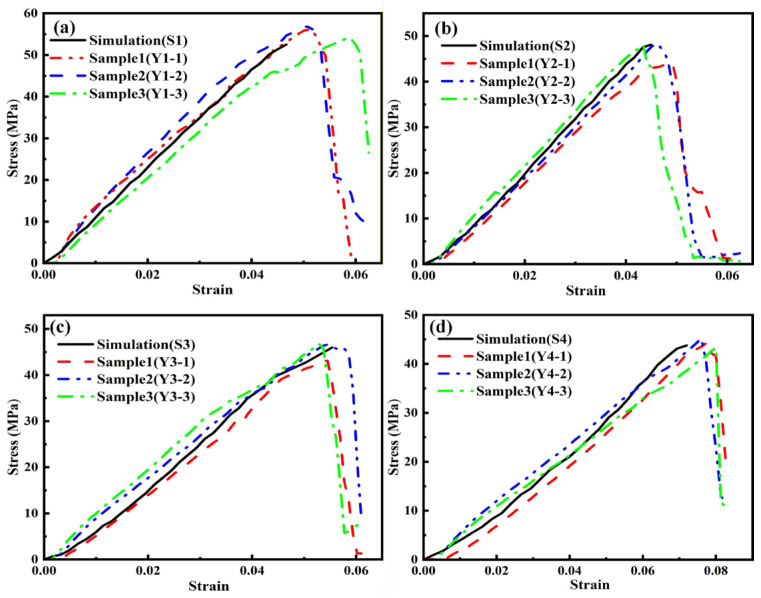
Comparison chart of simulation data and tensile test data (tensile condition: a yarn length of 50 mm and tensile speed of 1 mm/s) for 4 types of yarn: (**a**) S1, (**b**) S2, (**c**) S3, and (**d**) S4.

Figure 14 compares the simulation results of stress–strain curves of four types of yarns (the friction coefficient of 0.5) with experimental testing results. Obviously, it can be seen that the slopes of stress–strain curves of all yarns obtained from the numerical simulations and experimental tests (tensile condition: a yarn length of 50 mm and tensile speed of 1 mm/s) have a slight discrepancy, indicating that there is a slight difference of the elastic modulus obtained between the simulation and experiment.

### 4.3. Tensile Modulus Analysis

**Figure 15:**
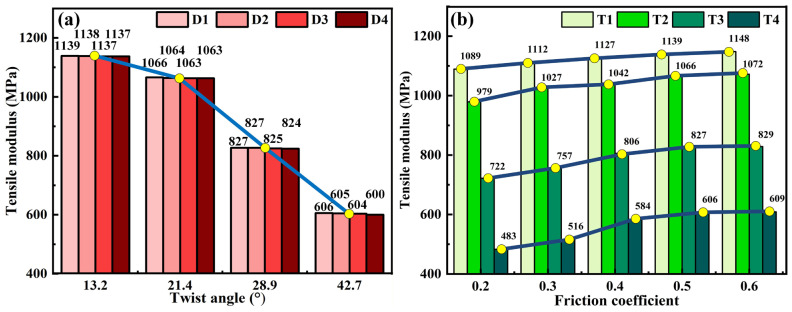
The effects of yarn diameter, twist angle, and friction coefficient on the tensile modulus of yarn: (**a**) the coupling effect of yarn diameter and twist angle, and (**b**) the coupling effect of twist angle and friction coefficient.

In this section, the effects of yarn diameter, twist angle, and friction coefficient on the tensile modulus of yarn are studied and presented in Figure 15. Figure 15a shows the coupling effect of yarn diameter and twist angle on the tensile modulus of yarn with an identical friction coefficient of 0.5, in which the corresponding yarn diameters D1, D2, D3 and D4 are 0.105 mm, 0.135 mm, 0.165 mm and 0.195 mm, respectively. The tensile modulus of yarn is nearly identical at the same twist angle, indicating that the yarn diameter has a negligible influence on the tensile modulus. This is because the tensile modulus is the intrinsic resistance of yarn to the tensile deformation, independent of its dimensional characteristics. Furthermore, with the increase in twist angle, the tensile modulus of yarn decreases significantly, especially when the twist angle reaches 28.9°. Similar results have been reported in the literature [26]. Figure 15b presents the coupling effects of twist angle and friction coefficient on the tensile modulus of yarn with an identical yarn diameter of 0.105 mm, in which the corresponding twist angles T1, T2, T3 and T4 are 13.2°, 21.4°, 28.9°, and 42.7°, respectively. It can be obviously found that the tensile modulus of yarns with different twist angles increases slightly with the increase in friction coefficient. The reason for this phenomenon is that fiber slippage is more likely to occur at a low friction coefficient when the yarn is stretched, and the low friction force between fibers causes the low tensile resistance of yarn. At a high friction coefficient, the friction force between fibers increases and makes the slip between fibers more difficult and the load distribution among the fibers more uniform, leading to a high tensile load and tensile modulus. From Figure 15, it can be concluded that twist angle and friction coefficient are two key factors affecting the tensile modulus of yarn.

**Figure 16:**
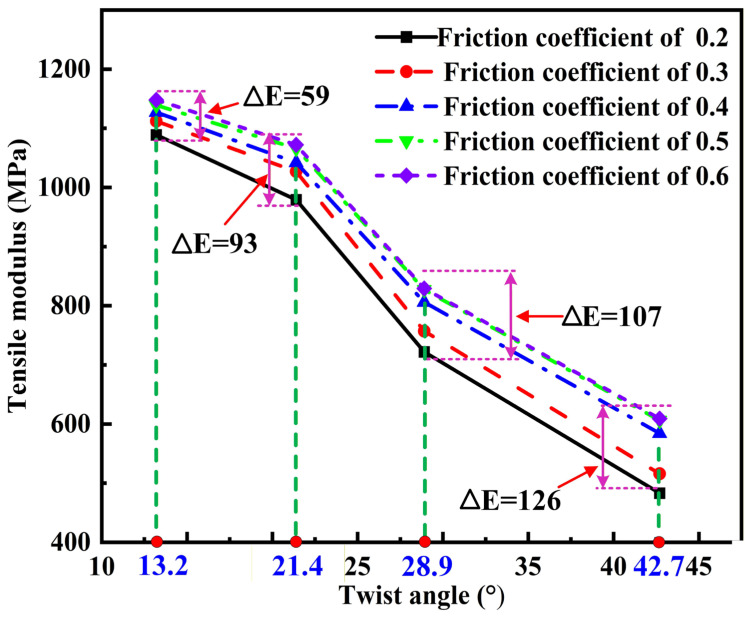
Comparison chart of tensile modulus for different twist angles and friction coefficients in the yarn with a diameter of 0.105 mm.

The difference of tensile modulus of yarn affected by the combined effects of twist angle and friction coefficient is further studied and shown in Figure 16. It can be observed that the effect of friction coefficient on the tensile modulus of yarn with a higher twist angle is more significant. For the yarn with a high twist angle of 42.7°, the tensile modulus increases markedly when the friction coefficient increases to 0.4 and then almost remains constant, which suggests that a friction coefficient of 0.4 is sufficient to provide the ‘mutual locking’ of the yarn structure with a high twist. In the actual production of twisted yarn, a high friction coefficient can be obtained by increasing the twist level [24]. Similar results have been reported in the work of Zhang et al. [35]. For the yarn with a low twist angle of 13.2°, the tensile modulus increases slightly with the increase in friction coefficient. This is because the high yarn twist causes the radial pressure between fibers to grow exponentially, substantially increasing the influence of the friction coefficient.

Combining the simulation results shown in Figure 15 and Figure 16, the tensile modulus of cotton yarn primarily depends on the elastic properties of fibers, the yarn twist level, and the friction coefficient between fibers. The ‘rule-of-mixtures’ equation had been proposed by Miao et al. to exemplify the relationship between the tensile modulus of yarn and the yarn twist level and the elastic properties of fibers, which is presented as follows [24]:

(1) $$E_{y}=\eta_{0}V_{f}E_{f}$$

(2) $$\eta_{0}=\cos^{2}\;\theta$$

where *E_y_* is the tensile modulus of yarn; *E_f_* denotes the elastic modulus of fiber; *η*_0_ is a factor related to fiber orientation; *V_f_* is the volume fraction of fiber within the yarn and *θ* is the surface twist angle of yarn.

**Figure 17:**
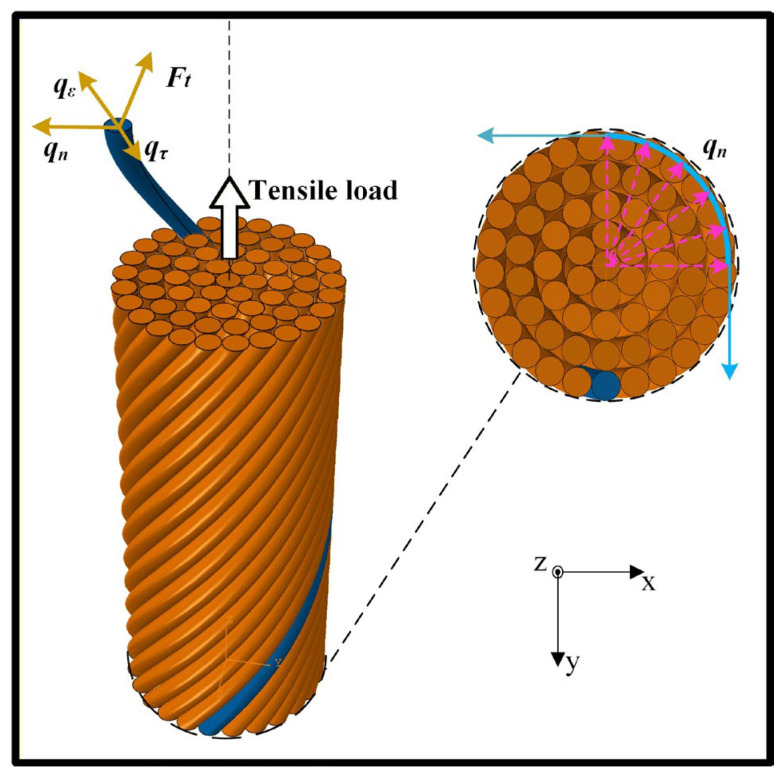
Load analysis of fibers within the yarn under a tensile load.

It is obvious that the effect of friction between fibers is not taken into account in Equation (1), which is a key factor affecting and increasing the tensile modulus of yarn. Figure 17 illustrates the load condition of fibers within the yarn under a tensile load along the axial direction of the yarn. As shown in Figure 17, the tensile load (*F_t_*) provides the elastic deformation load (*q*_ε_) of a single fiber and resists the frictional load (*q_τ_*) between fibers along the tangent direction of the fiber helix.

The normal contact force *q_n_* between the fibers had been proven to be directly proportional to the square of sin *θ* in the literature [39], which can be written as follows:

(3) $$q_{n}\propto \sin^{2}\;\theta$$

According to the Coulomb’s law of friction [40], the relationship between the tangential contact force *q_τ_* along the fiber main axis direction and the normal contact force *q_n_* and the friction coefficient *μ_f_* can be given in the following Equation (4).

(4) $$q_{\tau }=\mu_{f}q_{n}$$

Therefore, the tangential contact force *q_τ_* between the fibers is also directly proportional to the square of sin *θ* and the friction coefficient *μ_f_*, shown as follows.

(5) $$q_{\tau }\propto \mu_{f}\sin^{2}\;\theta$$

Based on the aforementioned load analysis of fibers within the yarn under a tensile load and Equations (1) and (2), a modified ‘rule-of-mixtures’ equation to calculate the tensile modulus of twisted yarn is proposed through introducing the friction correction factor *η_μ_*, as shown in Equations (6) and (7).

(6) $$E_{y}^{\prime }=\eta_{0}\eta_{\mu }V_{f}E_{f}$$

(7) $$\eta_{\mu }=\left(1+\alpha \mu_{f}\sin^{2}\;\theta \right)$$

where $E_{y}^{\prime }$ is the modified tensile modulus of yarn; *η_μ_* is the friction correction factor; *α* is the correction factor, which is related to the contacting state of cotton fibers and takes into account the effect of shear force in the non-fiber main axis direction. The value of correction factor *α* is over 1 because the shear force between fibers is composed of the fiber main axis direction and non-fiber main axis direction. Because of the relatively slight shear force in the non-fiber main axis direction under the small strain tensile testing and simulation, the *α* is set to 1 here.

**Figure 18:**
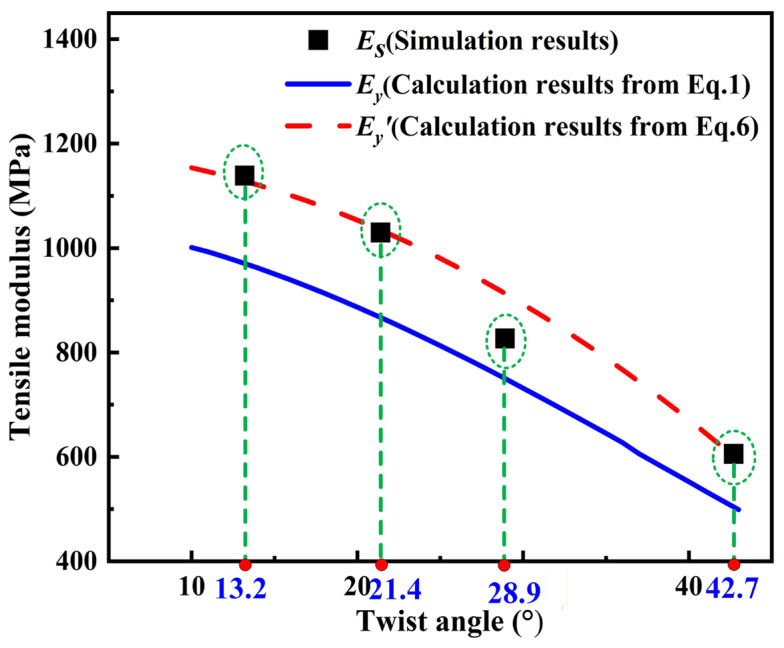
Comparison of simulation results and theoretical calculations for tensile modulus of yarns with four different twist angles at a friction coefficient of 0.5 and diameter of 0.105 mm.

In order to verify the accuracy of the modified ‘rule-of-mixtures’ equation, Figure 18 compares the prediction results of tensile modulus of yarn calculated from Equations (1) and (6) with those obtained from FE simulations with a friction coefficient of 0.5, which are most consistent with the experimental results. It can be clearly found from Figure 18 that the prediction results of Equation (6) are more accurate than those of Equation (1), verifying the accuracy of the modified ‘rule-of-mixtures’ equation.

## 5. Conclusions

In this paper, the tensile behavior of cotton ring-spun yarn with twisted staple fibers is investigated through experimental testing, numerical simulation, and theoretical calculation. Basic finite element models of yarns are developed according to the microscopic geometric morphologies of four types of yarn. Tensile experiments are conducted firstly to study the effects of tensile speed and yarn length on the tensile properties of four types of yarn. Finite element simulations are further performed to investigate the influence of yarn diameter, twist angle, and inter-fiber friction coefficient on the tensile modulus of yarn. Finally, a modified ‘rule-of-mixtures’ equation is proposed to calculate the tensile modulus of twisted yarn through introducing the friction correction factor. Based on the studies, the following conclusions are drawn:

1. Under different tensile experimental conditions, the yarn (Y1) with the smallest diameter of 0.112 mm and the lowest twist angle of 13.2° exhibits the highest tensile modulus and strength, while the yarn (Y4) with the largest diameter of 0.208 mm and the highest twist angle of 42.7° presents the lowest tensile modulus and strength. The tensile modulus and strength of four tested yarns are slightly affected by the yarn length and tensile speed.
2. The fitting error between the tensile moduli of all yarns obtained from numerical simulation with a friction coefficient of 0.5 and those obtained from the experimental testing is less than 2%. In addition, the simulated stress distribution of yarns S1 and S2 with a relatively smaller yarn diameter and lower twist angle under a tensile load is more uniform, which contributes to their higher tensile strength and modulus.
3. Based on the simulation results of idealized FE models, the twist angle and friction coefficient are two key factors affecting the tensile modulus of yarn. Furthermore, the yarn diameter has a negligible influence when the twist angle is held constant. The tensile modulus of yarn decreases significantly with the increase in twist angle, especially when the twist angle reaches 28.9°. The tensile modulus of the yarn with a low twist angle of 13.2° increases slightly with the increase in friction coefficient, while the tensile modulus of yarn with a high twist angle of 42.7° increases markedly when the friction coefficient reaches 0.4 and then almost remains constant, suggesting that a friction coefficient of 0.4 for the friction between cotton fibers is sufficient to provide the ‘mutual locking’ of the yarn structure with a high twist.
4. The tensile modulus calculated by the modified ‘rule-of-mixtures’ is more accurate compared with that calculated by the unmodified ‘rule-of-mixtures’, indicating that the modified ‘rule-of-mixtures’ equation through introducing the friction correction factor presents a higher accuracy for the prediction of tensile modulus of yarn.
5. The work provides theoretical guidance for the performance analysis and industrial application of cotton ring-spun yarn. In addition, the numerical modeling method and the modified ‘rules of mixtures’ can be applied to the mechanical property analysis of yarn with twisted fibers and composites made of twisted yarn, offering valuable insights into material constitutive behavior.

## Figures and Tables

**Table 1:** Yarn parameters.

Yarn Type	Yarn Average Diameter (mm)	Tex	Twist (Turns·cm^−1^)
Y1	0.112	14.8	6.7
Y2	0.146	18.5	8.5
Y3	0.169	28.1	10.4
Y4	0.208	36.9	14.1

**Table 2:** Parameters of yarn simulation model.

Type	Yarn Diameter (mm)	Twist Angle (Degrees)	Fiber Diameter (μm)	Yarn Twist Turns	Yarn Length (mm)	Number of Fibers	Number of Layers
S1	0.105	13.2	15	1	1.86	37	4
S2	0.135	21.4	15	2	2.31	62	5
S3	0.165	28.9	15	3	2.19	82	6
S4	0.195	42.7	15	4	2.00	102	7

**Table 3:** Basic mechanical properties of cotton fiber [18,37].

Property	Value
Longitudinal modulus, *E*_1_ (MPa)	1520
Transverse modulus, *E*_2_ (MPa)	196.73
Out-of-plane modulus, *E*_3_ (MPa)	196.73
Shear modulus, *G*_13_ = *G*_13_ (MPa)	85.288
Shear modulus, *G*_23_ (MPa)	56.192
Poisson’s ratio, *υ*_12_ = *υ*_13_	0.3
Poisson’s ratio, *υ*_23_	0.32

**Table 4:** Five element meshing plans of cotton fibers.

Meshing Plan	Element Size	
	Meshing Along the Length Direction (*L*_1_/μm)	Meshing of Cross Section (*L*_2_/μm)
1	40	2.9
2	20	3.8
3	20	2.9
4	10	2.9
5	20	1.9

## Data Availability

The original contributions presented in this study are included in the article. Further inquiries can be directed to the corresponding authors.
